# Recent Developments in Electrochemical-Impedimetric Biosensors for Virus Detection

**DOI:** 10.3390/ijms232415922

**Published:** 2022-12-14

**Authors:** Zala Štukovnik, Urban Bren

**Affiliations:** 1 Faculty of Chemistry and Chemical Engineering, University of Maribor, Smetanova ulica 17, 2000 Maribor, Slovenia; 2 Faculty of Mathematics, Natural Sciences and Information Technologies, University of Primorska, Glagoljaška ulica 8, 6000 Koper, Slovenia; 3 Institute for Environmental Protection and Sensors, Beloruska ulica 7, 2000 Maribor, Slovenia

**Keywords:** electrochemical impedance spectroscopy, impedimetric biosensor, genosensor, aptasensor, immunosensor, virus detection, SARS-CoV-2, HIV, influenza virus, hepatitis virus

## Abstract

Viruses, including influenza viruses, MERS-CoV (Middle East respiratory syndrome coronavirus), SARS-CoV (severe acute respiratory syndrome coronavirus), HAV (Hepatitis A virus), HBV (Hepatitis B virus), HCV (Hepatitis C virus), HIV (human immunodeficiency virus), EBOV (Ebola virus), ZIKV (Zika virus), and most recently SARS-CoV-2 (severe acute respiratory syndrome coronavirus 2), are responsible for many diseases that result in hundreds of thousands of deaths yearly. The ongoing outbreak of the COVID-19 disease has raised a global concern and intensified research on the detection of viruses and virus-related diseases. Novel methods for the sensitive, rapid, and on-site detection of pathogens, such as the recent SARS-CoV-2, are critical for diagnosing and treating infectious diseases before they spread and affect human health worldwide. In this sense, electrochemical impedimetric biosensors could be applied for virus detection on a large scale. This review focuses on the recent developments in electrochemical-impedimetric biosensors for the detection of viruses.

## 1. Introduction

In the last decade, several biosensors have been developed as an alternative method for the analysis of microorganisms, viruses, and toxins in food, as well as for various environmental and medical applications due to their ability of rapid analysis, reproducibility, stability, and accuracy [1,2,3]. As viral diseases currently threaten human health, the pathogen detection has emerged as one of the most relevant aims of biosensing devices [4,5].

Viruses have excellent resistance and high transmissibility, as well they can mutate rapidly and recombine their genetic material, which increases the likelihood of a pandemic, especially in a globalized world [6]. Numerous viruses such as influenza viruses, human immunodeficiency virus (HIV), Ebola virus (EBOV), Zika virus (ZIKV), or coronaviruses have significantly affected public health from the smallpox epidemic in the Aztec Empire in 1520 to the current pandemic COVID-19 [7]. With the latest health-threatening pandemic in 2019, rapid and sensitive detection of such pathogens has become even more critical [8].

Conventional methods, including an enzyme-linked immunosorbent assay (ELISA) and a polymerase chain reaction (PCR) are commonly applied to detect viruses such as the influenza viruses, Middle East respiratory syndrome coronavirus (MERS-CoV), severe acute respiratory syndrome coronavirus (SARS-CoV), human immunodeficiency virus (HIV), Ebola virus (EBOV), Zika virus (ZIKV), and severe acute respiratory syndrome coronavirus 2 (SARS-CoV-2) [9,10,11]. These traditional methods exhibit several drawbacks, including complex and laborious sample preparation and expensive equipment [12,13]. The rapid, sensitive, and on-site detection methods are critical for diagnosing and treating infectious diseases, including viruses before they spread and affect human health worldwide. In this sense, electrochemical-impedimetric biosensors could be massively applied to detect viruses [1].

By integrating a biological recognition component with a transducer, which transforms the activity of the biorecognition element into a measurable signal, biosensors represent valuable diagnostic tools for analyzing biological samples [2,14,15,16,17]. Electrochemical biosensors can be voltammetric, potentiometric, conductometric, amperometric, impedimetric, polarographic, capacitive, or piezoelectric, depending on the detection principle and application [18]. Electrochemical impedimetric biosensors that combine impedance and biorecognition elements have been widely used in virus detection in recent years [19,20].

This review focuses on the recent development of electrochemical impedance immunosensors and DNA- or RNA-based biosensors to detect currently circulating viruses.

## 2. Electrochemical Impedance Spectroscopy (EIS)

Electrochemical impedance spectroscopy (EIS) represents an emerging electrochemical technique [21,22]. In the field of biosensors, EIS is used to characterize the transduction of biosensing events at electrodes as well as biocatalytic and electrode transformations [8,23,24].

The EIS is based on the frequency dependence response of an electrochemical system to a small amplitude sinusoidal voltage signal and integrates the information about the capacitive and resistive properties of materials [25,26,27]. This method has been successfully applied to observe immunological bindings events such as antigen with antibody interaction at the electrolyte interface or the electrode, the synthesis of materials, as well as to toxicological studies monitoring changes in cell motility and morphology [28,29,30,31]. It also represents an advantageous technique for biosensor development since it is a non-destructive method that provides high-quality data [32]. In addition, the EIS system setup is small and portable. Therefore, analysis can also be performed outside the central laboratory [32,33].

The principle of the EIS method is a sinusoidal potential in the potenciostatic EIS, or current in the galvanostatic EIS, is employed in an investigated electrochemical system, where the resulting current or the potential is monitored in the frequency dependence [34,35]. The quotient of the potential *E*(ω,t) and the current I(ω,t) is the impedance *Z*(ω,t) (Equation (1)). In the equation, ω represents the angular frequency, t represents the time, *i* represents the imaginary number, and *φ* represents the phase angle between the potential or current signals [34].

(1) $$Z\left(\omega t\right)=\frac{E\;\left(\omega t\right)}{I\;\left(\omega t\right)}=\left|Z\left(\omega t\right)\right|e^{i\varphi }=\left|Z\left(\omega t\right)\right|\left(cos\varphi +isin\varphi \right)=Re\left(Z\right)+i\cdot Im\left(Z\right)$$

The EIS measurements can be performed with various electrodes in different configurations, the most common of which is usually referred to as two-, three-, and four-electrode systems. In practice, the measurement becomes more complex and precise with a higher number of electrodes [36,37,38]. Electrochemical impedimetric biosensors typically utilize the three-electrode configuration. This electrochemical cell configuration includes the reference (RE), the working (WE), and the counter electrode (CE) [37,38,39]. An impedimetric biosensor for virus detection has immobilized proteins specific for a particular virus, a viral genome’s complementary probe, or virus-specific antibodies to detect viral proteins, genomes, or antigens at the WE [38].

The presence or absence of redox species in the electrode or the electrolyte can determine whether the EIS is faradaic or non-faradaic. The EIS is faradaic if the redox species are present; otherwise, the EIS is non-faradaic. [34,40]. Choosing the most applicable method depends primarily on the predicted application [34]. In the faradaic category, the impedance is generated by redox reactions, while the non-faradaic category represents an impedance based on direct current (DC), whose electrical properties are caused by the double-layer capacitance [34,39,41]. Although the non-faradaic techniques offer the advantage of application in point-of-care devices due to the ability to miniaturize the electrodes and the absence of a redox couple, the faradaic sensors tend to be more sensitive and are typically applied more frequently in virus detection [34]. Due to its stability in aqueous solutions, reversible heterogeneous kinetics as well as well-defined redox processes, the [Fe(CN)_6_]^3−/4−^ is often used as a redox pair in the faradaic EIS, where the generation of electric current results from reduction or oxidation reactions among the electroactive species [6,34]. Moreover, the low oxidation potential of redox probes can reduce or avoid the occurrence of interfering species, which is particularly important for the analysis of real samples [6,34,42].

**Figure 1:**
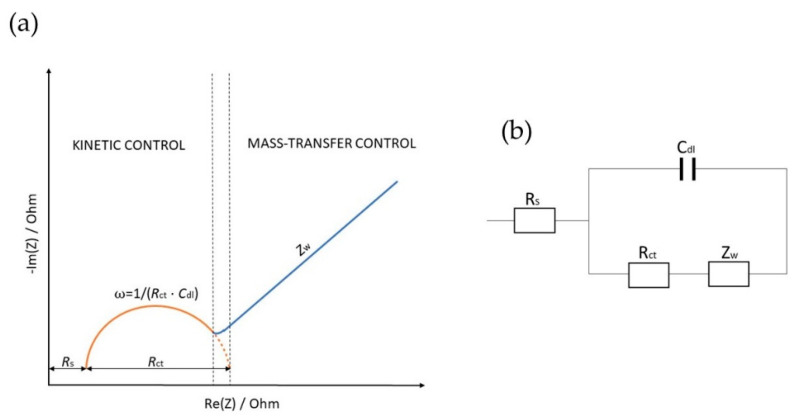
Representation of the Nyquist diagram (**a**) with the interrelated equivalent electric circuit EEC (**b**).

Z(*i* ω,t) is mainly measured across a wide frequency range since different components in heterogeneous materials may have differing mobilities [36]. Experimental values of the EIS measurements are usually plotted using the Nyquist plot, where the *Im*(*Z*) is plotted against the *Re*(*Z*) for each frequency [36,43]. Since Nyquist plots represent impedance dependence on frequency, data are sometimes also represented using Bode plots, where the impedance (|*Z*|) and the phase angle (*φ*), or alternatively the *Re*(*Z*) and the *Im*(*Z*), are plotted against frequency (ω) on a logarithmic scale [36]. Nyquist plots typically consist of the semicircular and the linear part (Figure 1a), which relate to the modifying layers of the electrode [44,45]. The semicircular part detected at higher frequencies describes the electron transfer, and the linear portion describes the diffusion-limited process [45,46]. Moreover, from the semicircle diameter, the charge transfer resistance (*R*_ct_) at the electrode surface is calculated, which usually increases after the virus, protein, or biomarker has bound to the biorecognition element [44,45].

Equivalent electric circuits (EEC) are typically used to interpret the data. Although more complicated components such as constant phase elements or Warburg impedances can be included, EEC primarily consists of resistors and capacitors [47]. An EEC is modeled after the sensing region [48]. From the measured impedance and phase angle data, the values of the fitted circuit elements are extracted to monitor changes in system behavior [48]. The Randles–Ershler EEC model represents the most common EEC for a simple electrochemical reaction (Figure 1b) [49]. This EEC consists of an ohmic resistance (*R*_s_), representing the resistance of the electrolyte solution between WE and RE, charge transfer resistance (*R*_ct_), the double-layer capacitance (*C*_dl_) describing the capacitance of the complex biological active layer, and the Warburg impedance (*Z*_w_), describing the normal diffusion through the complex biological active layer to the electrode surface [50,51,52].

The EEC in Figure 1b corresponds to the Nyquist diagram in Figure 1a. At low-frequency values, the main effect represents the ion diffusion named Warburg impedance, represented by a 45° straight line. The plot at high frequencies is primarily described by a semicircle whose diameter is determined by an *R*_ct_ [53]. In virus detection, *R*_ct_ is most commonly utilized to estimate the viral concentration. When the viruses bind to their target receptors on the surface of the WE, the redox reaction is inhibited, resulting in an *R*_ct_ increase [54].

## 3. Viruses

Viruses, including influenza viruses, MERS-CoV, SARS-CoV, Hepatitis A virus (HAV), Hepatitis B virus (HBV), HIV, EBOV, and most recently, SARS-CoV-2, are responsible for causing various diseases, and taking hundreds of thousands human lives yearly [6,55,56,57].

The location of a virus and the type of cells it affects determine the disease it will cause [58]. Viruses represent obligate intracellular parasites. Thus, they must invade a cell to replicate [59]. The virus is composed of a protein capsid encasing a genome (DNA or RNA), and, in the case of the majority of mammalian viruses, a lipid envelope surrounds the capsid [60]. 

It Is essential for viruses to enter cells since they can only replicate within cells [61]. Therefore, viral proteins are often expressed together with the envelope, facilitating recognition and binding to specific cells [38]. A virus must initiate its specific binding to the host cell to infect a cell [62]. The virus contains a virus attachment protein that binds to a cell surface receptor on the cell [63,64]. Influenza viruses contain hemagglutinin (HA) and neuraminidase (NA) as surface proteins that induce attachment to sialic acid residues on various mammalian cells [55,65]. HA and NA virus surface proteins are used to categorize influenza A viruses (H1N1, H3N2, etc.,), distinguishing 18 HA and 11 NA subtypes [66,67]. The HIV protein envelope binds to the primary cellular receptor cluster of differentiation 4 (CD4) and then to a cellular coreceptor (CCR5 or CXCR4) to infect cells [68]. The infection is initiated by this sequential binding, which activates the binding of the viral particles to the host cell membranes [63,69]. To infect cells, EBOV uses T-cell immunoglobulin mucin domain-1 (TIM-1) as its receptor [70,71]. The cellular receptor dipeptidyl peptidase 4 (DPP4) is targeted by the MERS-CoV’s spike glycoprotein (S). Coronaviruses are named after the crown-like spike glycoprotein S [72,73]. This glycoprotein has S1 and S2 subunits on the envelope [72]. SARS-CoV-2 binds with the S protein to the angiotensin-converting enzyme 2 (ACE2) [74,75]. The S2 subunit promotes fusion and entry of the virus into the host cell, while the S1 subunit of the S protein has a receptor-binding domain (RBD) that has a high binding affinity for the ACE2 receptor on human cells [76]. The immune system of SARS-CoV-2-infected individuals responds to the highly immunogenic S protein with the production of neutralizing antibodies and T-cell responses [76,77,78,79]. The ACE2 also represents the prime receptor of SARS-CoV [80,81].

## 4. Electrochemical Impedimetric Biosensors for Virus Detection

**Figure 2:**
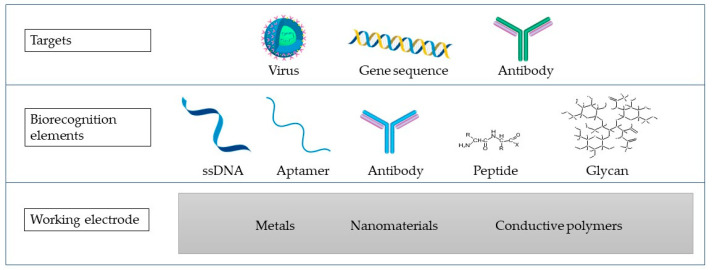
A biosensor scheme containing the WE made of different materials (metals, nanomaterials, or conductive polymers), biorecognition elements, and the targets that are commonly targeted in virus detection using the impedimetric biosensors.

Electrochemical biosensors represent biosensing devices that contain an electrochemical transducer that converts biochemical information with high sensitivity into a measurable signal [18]. They also possess advantages such as time-saving, simple instrumentation, and cost-effectiveness [18,82]. Biosensors contain a bioreceptor that specifically responds to the analyte, linked to an interface and an element of signal transduction that translates the binding of the analyte into a measurable signal [2,17]. Different electrochemical biosensors can be developed to identify and quantify viruses depending on the integrated biological component (Figure 2). These sensors can be generically classified as immunoassays and DNA- or RNA-based assays such as genosensors and aptasensors. Comparing the application of immunoassays to DNA- or RNA-based assays depends on a variety of parameters. These variables include the infection stage, the antibody’s availability, and data on DNA or RNA sequences [5].

Affinity sensors, which use selective binding of biomolecules, including antibodies, membrane receptors, or oligonucleotides with the analyte of interest to produce a quantifiable electrical signal, are the most often used biosensors for viral detection [83,84]. Generally, in affinity biosensors, the target analyte’s complementary binding site size and shape determine molecular recognition [84]. Thermodynamic considerations control the binding process, including DNA hybridization and antibody–antigen complexation [84]. Immobilized antibodies, antigens, and nucleic acids are the most common biorecognition elements used in the scientific literature to detect viruses [85]. Immobilization represents either a physical or a chemical process in which the entire biological recognition element is entrapped or there is an interaction of its portion with the surface of the transducer [86]. There exist four main types of immobilization, including adsorption and encapsulation, which belong to physical methods, as well as crosslinking and covalent bonding, which belong to chemical immobilization methods [87]. The selection of a suitable immobilization technique represents one of the crucial steps in the preparation of a biosensor, since the inactivation of the biological recognition element due to the choice of an inappropriate immobilization method is likely [88]. The most common immobilization strategies in the development of biosensors for virus detection consist of physical adsorption, covalent bonding, entrapment, and affinity-based interaction [89].

The detection principles employed in biosensors can be divided into label-based and label-free [90]. In label-free biosensors, the measurable signal is generated from a transducer, corresponding to the biorecognition event between the analyte of interest and the correlating receptor [91]. Sandwich assays are typical examples of label-based biosensors. In a sandwich assay, the analyte is captured by a receptor, such as an antibody, immobilized over the biosensor. The captured analyte attaches to the secondary receptor, such as a secondary antibody, which is then labeled with a fitting molecule to provide the measurable signal [91,92]. Due to their ability to be mass-produced at low-cost, electrochemical techniques have recently attracted much attention in the biosensor development [17,39]. In this aspect, EIS represents an essential technique for studying and comprehending the interfacial characteristics associated with particular biorecognition events, including the capture of antigen antibodies at the electrode surface or the molecular biorecognition of specific proteins, the identification of receptors, nucleic acids, or even whole cells [3,39].

However, there remain several limitations that need to be overcome. One of them is specificity, which is considered the most important property of a biosensor, as it describes the ability of a sensor to distinguish between target and non-target biological components of a sample [93]. Moreover, an unavoidable problem is the cross-talk between electrochemical and electrophysiological signals [94]. For example, some viral proteins share a certain sequence identity with other viral species (e.g., the envelope, nucleocapsid, membrane, and spike proteins of SARS-CoV-2, SARS-CoV, and MERS-CoV) [95]. To overcome these limitations, care must be taken in selecting biorecognition elements specific to each virus to reduce the cross-reactivity and to avoid false positive results [96]. Moreover, the use of biological receptors in biosensors has well-known limitations including low stability of the biological species, as well as low chemical and thermal stabilities [30]. The stability of the electrodes also plays an important role in the development of a biosensor. Electrodes made of Au are most commonly used, as they are both biocompatible and stable [19]. Compared to optical biosensors, where diagnostics are based on a sensitive detection of photon emission from dyes and other molecules excitable by light, impedimetric biosensors tend to have a lower sensitivity. However, unlike fluorescence and bioluminescence-based detection, electrochemical biosensors are easier to use with non-clear samples such as blood. In addition, electrochemical detection does not require a complex optical apparatus used in many fluorescence-based detections [97].

Several studies on impedimetric biosensors have been performed on designing the genosensors, the aptasensors, and the immunosensors.

**Figure 3:**
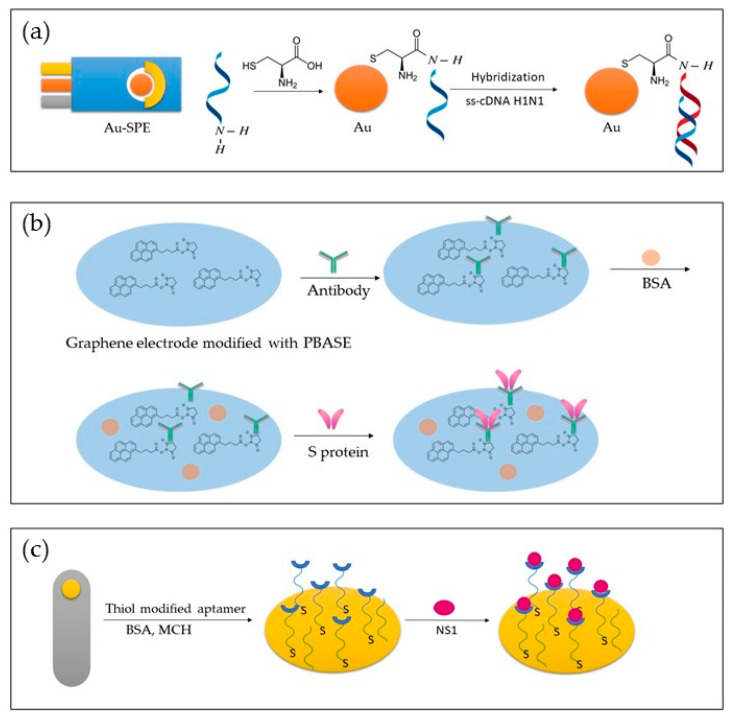
Representation of different approaches in the development of genosensors (**a**), immunosensor (**b**), and aptasensor (**c**).

Figure 3 depicts different approaches to biosensor development. In Figure 3a, a genosensor was developed using Au-SPE modified with cytosine. In Figure 3b, an aptasensor is presented, where a graphene electrode modified with PBASE was used to detect the S protein. In Figure 3c, a bare gold electrode was modified with thiol-modified aptamer, BSA and MCH, and NS1 was detected.

### 4.1. Genosensors for Virus Detection

One of the types of biosensors for virus detection receiving growing attention is the genosensor, which has been successfully applied for H1N1, HBV, EBOV, ZIKV, and HIV detection. A hybridization reaction between the DNA or RNA target and the ss-DNA sensing element in the genosensors allows for the detection of DNA or RNA targets [98,99]. The principle of detection with genosensors relies on the DNA or the RNA strand (probe) immobilization on the surface of a transducer to bind its complementary (target) sequence [60]. As the conventional biosensor assembly depends on single-strand hybridization, which is a reversible process, employing RNA or DNA has an advantage as it offers regeneration of the transducer surface [60,100]. Additionally, genosensors have a low limit of detection (LOD) [60,100].

**Figure 4:**
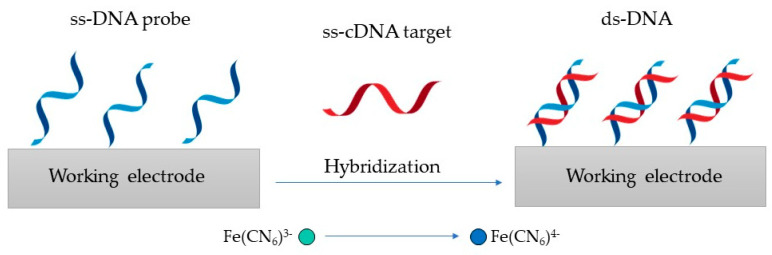
Genosensor principle, where the ss-DNA probe is hybridized with its ss-cDNA to produce ds-DNA.

The detection principle shown in Figure 4 is based on changes in the redox marker after the hybridization of the probe DNA with its complementary target DNA (ss-cDNA) [101].

In recent years, many new genosensors have emerged to detect various virus-related diseases and pathogens through the efforts of researchers (Table 1).

An impedimetric genosensor based on a HA gene sequence was devised by Ravina et al. [102]. In this study, an amino-labeled ss-DNA probe was immobilized onto the cysteine-modified gold surface of the screen printed electrode (Au-SPE) for detection of the H1N1 influenza strain in humans. Researchers recorded the electrochemical impedance spectrums after the hybridization of the probe with the H1N1 ss-cDNA in the presence of a redox couple with a frequency ranging from 0.1 Hz to 0.01 mHz. This study reported that the fabricated impedimetric biosensor could detect 0.004 ng ss-cDNA of H1N1 in 6 μL within only 30 min. 

Shariati and Sadeghi [103] devised a DNA biosensor for HBV detection, where EIS responses were biased under laser amplification. This biosensor was found on tin-doped WO_3_/In_2_O_3_ nanowires. The LOD of 1 fM was determined, where the corresponding *R*_ct_ values decreased from 2487 to 806 Ω for DNA complementary target and probe. The developed biosensor reportedly had a linear detection range from 0.1 pM to 10 μM.

A label-free impedimetric biosensor for the detection of HBV DNA based on ZnO nanowires doped with tellurium (Te) was devised by Khosravi-Nejad et al. [104]. This HBV biosensor detection range was in concentrations ranging from 1 pM to 1 μM, where the LOD of the developed genosensor was 0.1 pM.

Ilkhani and Farhad [105] fabricated an EBOV DNA biosensing device. In this study, a biotinylated target strand DNA was hybridized with a thiolated DNA capture probe sequence that was immobilized on the SPE surface. The LOD of complementary oligonucleotides was determined at 4.7 nM.

Moreover, a three-electrode and label-free impedimetric electrochemical DNA biosensor for the detection of ZIKV was reported by Faria and Zucolotto [106]. EIS measurements were performed with an alternating current (AC) perturbation, decreasing in frequency from 30 kHz to 0.1 kHz with ten measurement points per decade in a logarithmic scattering. Impedance measurements identified a LOD of 25.0 ± 1.7 nM. The linearity in measurements was achieved in the range from 54 to 340 nM.

An impedimetric HIV-1 genosensor was devised by Gong et al. [101]. This genosensor was developed by adsorbing ss-DNA onto the graphene-Nafion-modified surface of a glassy carbon electrode (GCE). Researchers explained in their study that as the negative ss-DNA adsorbs and the steric hindrance occurs, the *R*_ct_ of the electrodes toward the [Fe(CN)_6_]^3−/4−^ gets limited. In the process, the ss-DNA probe was hybridized with the target DNA to form ds-DNA. The helix formation induces ds-DNA release from the surface of the biosensor. The decrease in *R*_ct_ is logarithmically related to the concentration of the HIV-1 gene in a range from 0.1 pM to 100 nM. The LOD of this sensor is determined at 23 fM.

An alternative detection method for the HIV-1 gene using a label-free DNA impedimetric genosensor with gold nanoparticles (AuNPs)/carbonized glass fiber (GF) coal tar pitch electrodes (GTP) was designed by Yeter et al. [107]. The developed biosensor provided a LOD of 13 fM, with a linear range from 0.1 pM to 10 nM. Researchers used amine-crosslinking chemistry in preparation for the thiol-modified electrodes. In this study, the EIS with a frequency range from 100 to 0.1 kHz and a wave amplitude of 10 mV at a DC potential of 0.115 V was used for the determination.

### 4.2. Aptasensors for Virus Detection

Aptasensors are biosensors that use aptamers as biorecognition elements [108]. Aptamers represent short and synthetic single-stranded nucleic acids, either ss-DNA or ss-RNA [7]. Aptamers usually consist of lesser than 100 nucleotides, capable of selective binding onto a specific target [7]. Compared to genosensors, here, the DNA or the RNA aptamer plays the role of the receptor [98,109]. It is necessary to immobilize the aptamer strands and identify them to make detection easier when using aptamers in aptasensors. The preferred target for choosing virus-specific aptamers is either a protein produced from a virus or an inactivated virus particle [110]. The ss-DNA or ss-RNA oligonucleotide sequences used as the biorecognition element are screened in a SELEX (systematic evolution of ligands by exponential enrichment) procedure [84]. In a SELEX, the ability of ss-DNA or ss-RNA to selectively bind to low molecular weight organic, inorganic, or protein targets is screened [84,111]. Several studies have been performed on EIS-based biosensors, in which the aptasensors were developed (Table 2).

Kim et al. [112] devised a MERS-nanovesicle (NV) biosensor structured of multi-functional DNA aptamers and graphene oxide encapsulated molybdenum disulfide (GO-MoS_2_) hybrid nanocomposite. The electrical condition for an AC impedance measurement was a frequency ranging from 1 Hz to 100 kHz with an amplitude of 10 mV. The LOD of this biosensor was determined at 0.4049 pg/mL, and its linear range was from 70 pg/mL to 400 pg/mL.

Karash et al. [113] devised a label-based impedance aptasensor for H5N1 detection employing a specific aptamer for the H5N1 influenza strain and a gold interdigitated microelectrode (Au-IDE). In this study, a biotin-labeled H5N1 aptamer was bound to immobilize streptavidin on the surface of the microelectrode. According to the researchers, polyethylene glycol was utilized to block the microelectrode, and the attached aptamer captured the virus. Using a sinusoidal AC potential of 10 mV and a frequency range of 10 Hz to 1 MHz in the presence of [Fe(CN)_6_]^3−/4−^, the magnitude and phase of the impedance were measured at 54 points per decade. The LOD was determined at 0.25 HAU, and the linearity range was obtained from 0.125 to 16 HAU.

An electrochemical aptasensor for the detection of the HCV core antigen was developed by Ghanbari et al. [114]. In this study, the immobilization surface was prepared by the modification of a GCE with graphene quantum dots (GQD). With a 3.3 pg/mL LOD and a linear concentration range from 70 to 400 pg/mL, the EIS approach was used as a reliable detection technology for HCV core antigen.

A design of an aptamer-based viability impedimetric sensor for viruses was presented by Labib et al. [115]. In this study, cell-SELEX was employed to select highly specific DNA aptamers for intact vaccinia virus (VACV) that were later self-assembled onto Au microelectrode to form impedimetric biosensors. It was found that the developed aptasensor was highly selective and, therefore could detect viable VACV particles with a LOD of 60 virions/L or 330 PFU in a linear range from 500 to 3000 PFU, as well as differentiate them from non-viable viruses. In this research, EIS was applied to monitor the binding of the proposed aptamer to the target VACV, which decreased the interfacial resistance and, consequently, the *R*_ct_ value. According to this study, this occurrence caused the aptamers to alter conformation after binding to VACV, allowing the [Fe(CN)_6_]^3−/4−^ to adhere to the electrode surface more freely.

Bachour Junior et al. [116] devised an electrochemical biosensor for non-structural protein (NS1) detection using DNA aptamers. NS1 is a relevant biomarker that is seen in high concentrations in the blood during the early stages of dengue virus (DENV) infection. In this study, a self-assembled monolayer by immobilizing Au electrodes with particular aptamers and 6-mercapto-1-hexanol (MCH) was produced. Researchers obtained EIS results with a 10 mV amplitude in the frequency range of 100 kHz to 100 mHz. The device achieved a LOD of 22 pg/mL with a linear range from 10 pg/mL to 1 g/mL.

### 4.3. Immunosensors for Virus Detection

In the impedimetric immunosensors, the antibodies that interact with the viral antigens are immobilized on the electrodes. Due to their promising applications in various fields, they have recently gained great interest. [117,118]. In impedimetric immunosensors, an electrical signal difference results from the kinetic binding of antibodies and their antigens to the electrode surface. As a result, *R*_ct_ is altered, corresponding to the amount of bound antigens [39].

In immonosensors, the most commonly used biological components are IgG antibodies, which are large Y-shaped glycoproteins produced by a host in reaction to the presence of a foreign molecule called an antigen [84,111].

**Figure 5:**
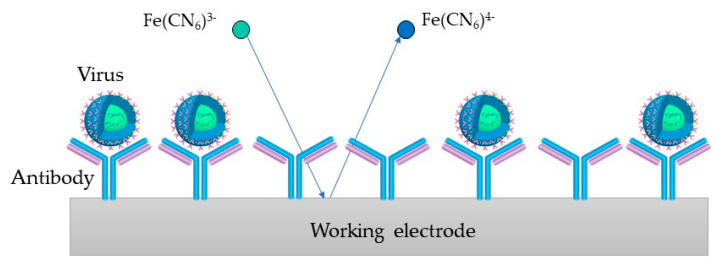
Immunosesor principle, where the virus binds to the antibody at the WE surface and the redox reaction gets hindered.

In Figure 5, the process on the WE containing antibodies as biorecognition elements is depicted (an immunosensor). [Fe(CN)_6_]^3−/4−^ is used as a redox probe in the process. The virus binds to the target bioreceptor (antibody) at the WE surface, and the redox reaction is hindered.

Several studies on EIS-based biosensors have been performed by designing immunosensors for virus detection (Table 3).

Dunajová et al. [119] developed a highly selective and ultra-sensitive impedimetric immunobiosensor for detecting influenza A viruses. The reported immunosensor was based on the interaction with monoclonal antibodies using a screen-printed carbon electrode (SPCE). Measurements in this study were performed at frequencies ranging from 0.05 Hz to 30 kHz. Antibodies and viral nucleoproteins were reported to change the layer thickness, resulting in an altered charge transfer resistance (Δ*R*_ct_). The biosensor was tested in an ideal buffered PBS solution where the LOD was 0.79 fM, and the linearity was obtained from 0.18 fM to 0.18 nM. 

Nidzworski et al. [120] devised a diamond biosensor for the influenza virus that enables specific virus detection at ultralow concentrations, even before any clinical symptoms appear. In this study, the M1 protein, a universal biomarker for influenza viruses, was identified by surface functionalizing a diamond electrode with polyclonal anti-M1 antibodies. A LOD of 1 fg/mL for the M1 biomarker in a saliva buffer, which corresponds to about 5 to 10 viruses per sample in 5 min, was reported.

Akkapinyo et al. [121] reported an impedimetric immunosensor for hepatitis B surface antigen (HBsAg) detection. This impedimetric immunosensor was developed by immobilizing hepatitis B surface antibody (Anti-HBs) through the N-ethyl-N0-(3-(dimethylamino)propyl)carbo-diimide/N-hydroxy succinimide (EDC/NHS) couple reaction, which involved the carboxyl group of the bovine serum albumin (BSA) cross-linked film on the SPCE. In this study, the scanning frequency was between 0.01 Hz and 100 kHz under an applied AC of 10 mV, where a linear relationship between Δ*R*_ct_ and HBsAg concentration was obtained in the range from 5 to 3000 ng/mL with a LOD of 2.1 ng/mL.

A label-based impedimetric biosensor was reported by Mandli et al. [122]. An indirect competitive electrochemical immunosensor for HAV detection was developed by immobilizing HAV antibodies on the carbon nanopowder paste electrode (CNPE) surface, using a secondary antibody labeled with peroxidase to target HAV antigen. The developed immunosensor provided exact data with a linear concentration range from 2 × 10^−4^ to 5 × 10^−3^ IU/mL, with the LOD at 26 × 10^−5^ IU/mL.

Chowdhury et al. [123] devised a biosensor where nanocomposites were deposited on an electropolymerized polyaniline-coated GCE to form an Ab-N,S-GQDs-AuNP-PAni/PAni||GCE sensor. HEV was then detected using an impedimetric response. The measurements were taken over a frequency range from 100 kHz to 100 mHz with an AC amplitude of 5 mV, where the LOD was determined at 8 fg/mL.

Kaushik et al. [124] presented an impedimetric immunosensor for ZIKV-protein detection. In this study, a functionalized interdigitated micro-electrode of gold (IDE-Au) was prepared by the immobilization of the ZIKV-specific envelope protein antibody (Zev-Abs). According to the findings of this EIS analysis, the biosensor selectively recognized ZIKV-protein in a linear detection range between 10 pM and 1 nM, with a LOD of 10 pM and a high sensitivity of 12 kΩ/M. 

Cabral-Miranda et al. [125] designed an immunosensor based on the recombination of domain III of the envelope protein (EDIII) and ZIKV non-structural protein 1 (NS1). Using EIS and squarewave voltammetry (SWV), it was demonstrated that the biosensor is sensitive to ZIKV-specific antibodies in serum and saliva and can immediately distinguish between ZIKV- and DENV-specific antibodies. This study performed EIS assays at a potential of 0.14 V, with an amplitude of 0.01 V and 50 frequency values logarithmically distributed from 0.1 to 100,000 Hz.

## 5. SARS-CoV-2 Detection

Currently, rapid and accurate diagnostic techniques are needed to prevent the further spread of COVID -19 disease worldwide. Due to this situation, many SARS-CoV-2 biosensors with different design protocols have been developed (Table 4).

Mojsoska and Larsen et al. [126] devised an electrochemical immunoassay label-free SARS-CoV-2 detection via S protein. The reported assay consists of graphene WE modified with anti-spike antibodies. The sensor has been reported to be able to detect a specific signal above 260 nM (20 μg/mL) of S1 of recombinant S protein and SARS-CoV-2 at a physiologically relevant concentration of 5.5 × 10^5^ PFU/mL.

Aydın et al. [77] developed an impedance sensing platform combined with conducting nanocomposites that have been applied to detect spike-receptor binding domain (RBD) proteins. This study synthesized a substituted thiophene monomer and electrodeposited it on the indium tin oxide (ITO) surface to produce a simplistic impedimetric biosensor. The constructed immunosensor had a LOD of 0.58 fg/mL, and a linearity range from 1.2 fg/mL to 120 pg/mL.

Zaccariotto et al. [127] devised a method for SARS-CoV-2 detection based on an impedimetric immunosensor using antibodies immobilized on the reduced graphene oxide (rGO). An electrochemical immunoassay was developed to detect S protein RBD using an impedimetric immunosensor and the redox probe [(Fe(CN)_6)_]^3−/4−^. The frequency ranged from 10 MHz to 0.01 Hz, and an amplitude of 10 mV was applied. The LOD was 150 ng/mL, and linearity from 0.16 to 1.25 μg/mL was obtained.

An EIS-based biosensor with a recombinant ACE2-coated palladium nano-thin-film (Pd-NTF) was devised by Kiew et al. [128] to screen for possible inhibitors of the S-protein-ACE2 binding. It was reported that this biosensor could detect interferences of small analytes with the S-protein-ACE2 binding at low analyte concentrations and small volumes with the LOD of 0.1 μg/mL. 

Lorenzen et al. [129] devised a modified electrode with electro-synthesized poly-(3,4-ethylene dioxythiophene) (PEDOT) and AuNPs. The truncated nucleoprotein (Naa160–406aa) was immobilized on the electrode. The reported approach involved employing [Fe(CN)_6_]^3−/4−^ to measure the *R*_ct_ before and after the modified electrode came into contact with the positive or negative serum sample. This investigation maintained the perturbation amplitude at 10 mV over a frequency range from 10 kHz to 10 mHz.

A genosensor for SARS-CoV-2 detection was reported by Avelino et al. [130]. In this study, a nanostructured platform of polypyrrole (PPy) and AuNPs was developed on miniaturized electrodes of tin-doped indium oxide (ITO). An oligonucleotide primer was chemically immobilized on the transducers for the biological detection of the nucleocapsid protein (N) gene. High selectivity was observed by not recognizing the biological targets in patient samples that were not infected with SARS-CoV-2. The devised biosensor had a LOD of 258.01 copies/µL and a linear response range of 800 to 4000 copies/µL.

A strategy for the detection of SARS-CoV-2 using interdigitated gold electrodes (AuIDE) with a spacing of 10 μm was presented by Ramanathan et al. [131]. In this study, the silane-modified AuIDE surface was deposited with a diamond, enhancing the detection of SARS-CoV-2 nucleocapsid protein (NCP). It was reported that EIS measurements were recorded at 100 mV AC amplitude, with a frequency range of 0.1 to 1 MHz. The LOD was determined at 0.389 fM. Moreover, good selectivity and a linear detection range from 1 fM to 100 pM were obtained. The detection of NCP in this study was evaluated by applying anti-NCP aptamer and antibody as the bioprobes.

Furthermore, in developing the impedimetric biosensor for SARS-CoV-2, an additional approach was taken using the peptides and matrix as biorecognition elements.

Soto and Orozco [132] devised a peptide-based impedimetric biosensor for simple monitoring of free S protein and SARS-CoV-2 viral particles in COVID-19 positive patients. This biosensor used a synthetic thiolated peptide bioreceptor chemisorbed at the WE of an Au-SPE. The thiolated peptide biosensors directly interacted with the S protein. In the evaluation, the developed device showed high sensitivity and reproducibility with a LOD of 18.2 ng/mL, and in commercial S protein solutions, the LOD was as low as 0.01 copies/mL in lysed SARS-CoV-2 particles. The linear range in this study was obtained from 0.05 to 1.0 μg/mL.

SARS-CoV-2 detection using a matrix as a biorecognition element was reported by Hussein et al. [133]. Researchers constructed an electrochemical biosensor using carbon nanotubes (CNTs) and tungsten trioxide (WO_3_) on the SPE to imprint the complete SARS-CoV-2 viral particles within the polymer matrix to create viral complementary binding sites. Measurements in this study were taken at an AC potential of 5 mV, with a frequency range from 10 kHz to 0.1 Hz. The developed biosensor exhibited high selectivity against the tested SARS-CoV-2 and other corona and influenza respiratory viruses. The LOD and limit of quantification (LOQ) were 57 and 175 pg/mL, and the linear range was obtained from 7 to 320 pg/mL.

## 6. Conclusions

Viral diseases, which result in more than one million new cases and hundreds of thousands of deaths each year, pose a severe threat to public health. In addition, viruses can change rapidly, making people vulnerable to emerging and potentially deadly viral strains, as evidenced by the COVID -19 pandemic currently affecting the entire world [60]. The present scenario relies on specific diagnostics such as polymerase chain reaction (PCR) and enzyme-linked immunosorbent assay (ELISA), which are high-priced and time-intensive [134]. Additionally, they are not available to the general public and can provide false-positive and false-negative results [60,135]. The point-of-care devices have gained increasing attention due to their advantages, such as high sensitivity, selectivity, reproducibility, low cost, and low sample quantity requirements, combined with a miniaturized device that is easy to handle and operate [136]. In this sense, impedimetric biosensors have been devised for rapid and on-site testing of various viruses.

In this review, recent developments in impedimetric electrochemical biosensors for the detection of various viruses such as influenza viruses, hepatitis viruses, HIV, ZIKA, EBOV, and coronaviruses are examined. Researchers have applied different approaches and inventive methods to develop these biosensors. With improvements in many areas, developed biosensing devices exhibit analytical performance comparable to conventional virus detection methods. One of their main advantages is their miniaturization ability, which enables the development of portable, adaptable, and low-sample consumption biosensors [6].

In summary, the determination of whole viruses and their components associated with a particular disease can be beneficial in differentiating and diagnosing diseases with similar clinical symptoms [6]. In addition, the latest progress in the development of biosensors for viral disease diagnosis has enabled quick and inexpensive in situ monitoring, even without complex and expensive equipment or a specialized workforce. Many challenges remain in the development and application of these biosensors. Nevertheless, the resulting devices show promise for real-time monitoring of specific viruses. Additionally, they are beneficial devices in pandemic scenarios as they facilitate sensitive and specific detection of pathogens with minimal resources.

## Figures and Tables

**Table 1:** Recently developed genosensors for virus detection.

Virus	Recognition Element	Target	Electrode	Linear Range	LOD	Reference
H1N1	ss-DNA H1N1	ss-cDNA H1N1 (HA)	Cysteine modified Au-SPE	/	0.667 ng/mL	[102]
HBV	ss-DNA HBV	ss-cDNA HBV	WO3/In2O3 nanowires	0.1 pM–10 µM	1 fM	[103]
HBV	ss-DNA HBV	ss-cDNA HBV	Te doped ZnO nanowires	1 pM–1 µM	0.1 pM	[104]
EBOV	ss-DNA EBOV	ss-cDNA EBOV	Au-SPE	/	4.7 nM	[105]
ZIKV	ss-DNA ZIKV	RNA (NS5 protein)	Au-PET	54–340 nM	25 nM	[106]
HIV	ss-DNA HIV	ss-cDNA HIV	Graphene-Nafion modified GCE	0.1 pM–100 nM	23 fM	[101]
HIV	ss-DNA HIV	ss-cDNA HIV	AuNPs/GF/CTP	0.1 pM–10 nM	13 fM	[107]

**Table 2:** Recently developed aptasensors for virus detection.

Virus	Recognition Element	Target	Electrode	Linear Range	LOD	Reference
MERS-CoV-2	MF DNA aptamer	MERS-NV	GO-MoS_2_	70–400 pg/mL	0.4049 pg/mL	[112]
H5N1	H5N1 aptamer	H5N1	Au-IDA microelectrode	16–0.125 HAU	0.25 HAU	[113]
HCV	HCV aptamer	HCV core antigen	GCE/GQD	10–70 pg/mL and 70–400 pg/mL	3.3 pg/mL	[114]
VACV	VACV aptamer	VACV particles	Au microlectrode	500–3000 PFU	330 PFU	[115]
DENV	DENV aptamer	NS1	MCH-Au electrodes	10 pg/mL–1 μg/mL.	22 pg/mL	[116]

**Table 3:** Recently developed immunosensors for virus detection.

Virus	Recognition Element	Target	Electrode	Linear Range	LOD	Reference
H3N2	Viral antibodies	Viral nucleoproteins	SPCE	0.18 fM–0.18 nM	0.79 fM	[119]
H1N1	M1-antibody	M1 protein	BDD	0–100 fg/mL	1 fg/mL	[120]
HBV	Anti-HBs	HBsAg	BSA-SPCE	5–3000 ng/mL	2.1 ng/mL	[121]
HAV	Anti-HAs	HAsAg	CNPE	2 × 10^−4^–5 × 10^−3^ IU/mL	6 × 10^−5^ IU/mL	[122]
HEV	Anti-HEV antibody	HEV	PAc-GCE	/	8 fg/mL	[123]
ZIKV	Zev-Abs	ZIKV-protein	IDE-Au	10 pM–1 nM	10 pM	[124]
ZIKV	Anti-NS1	NS1	SPCE	/	/	[125]

**Table 4:** Recently developed biosensors for SARS-CoV-2 detection.

Type of Sensor	Recognition Element	Target	Electrode	Linear Range	LOD	Reference
Immunosensor	S-RBD antibody	S-RBD protein	ITO	1.2 fg/mL–120 pg/mL	0.58 fg/mL	[77]
Immunosensor	S-RBD antibody	S-RBD protein	Graphene	/	20 μg/mL	[126]
Immunosensor	S-RBD antibody	S-RBD protein	rGO	0.16–1.25 μg/mL	150 ng/mL	[127]
Immunosensor	ACE2	S-RBD protein	Pd-NTF	/	0.1 μg/mL	[128]
Immunosensor	N protein (Naa160–406aa)	IgG	PEDOT-AuNPs	/	/	[129]
Genosensor	Oligonucleotide primer	N gene	ITO	800–4000 copies/µL	258.01 copies/µL	[130]
Aptasensor	N protein aptamer	N protein	AuIDE	1 fM–100 pM	0.389 fM	[131]
Peptide-based	Thiolated peptide	S-RBD protein	Au-SPE	0.05–1.0 μg/mL	18.2 ng/mL	[132]
Matrix	Polymeric matrix	virus particles	CNTs/WO_3_-SPE	7–320 pg/mL	57 pg/mL	[133]

## Abbreviations

AC	Alternating current
ACE2	Angiotensin-converting enzyme 2
Au-IDE	Gold interdigitated microelectrode
AuNPs	Gold nanoparticles
Au-SPE	Gold surface- screen printed electrode
BDD	Boron doped diamond
BSA	Bovine serum albumin
CD4	Cluster of differentiation 4
*C*_dl_	Double-layer capacitance
CE	Counter electrode
CNPE	Carbon nanopowder paste electrode
CNT	Carbon nanotube
COVID-19	Coronavirus disease 2019
DC	Direct current
DENV	Dengue virus
DPP4	Dipeptidyl peptidase 4
E	Envelope protein
EBOV	Ebola virus
EDIII	Domain III of the envelope protein
EEC	Equivalent electric circuit
EIS	Electrochemical impedance spectroscopy
ELISA	Enzyme-linked immunosorbent assay
GCE	Glassy carbon electrode
GF	Glass fiber
GO-MoS_2_	Graphene oxide encapsulated molybdenum disulfide
GQD	Graphene quantum dots
GTP	Tar pitch electrodes
HA	Hemagglutinin
HAV	Hepatitis A virus
HBsAg	Hepatitis B surface antigen
HBV	Hepatitis B virus
HCV	Hepatitis C virus
HIV	Human immunodeficiency virus
ITO	Indium tin oxide
LOD	Limit of detection
LOQ	Limit of quantification
M	Membrane protein
MCH	6-mercapto-1-hexanol
MERS-CoV	Middle East respiratory syndrome coronavirus
N	Nucleocapsid
NA	Neuraminidase
NCP	Nucleo capsid protein
NS1	Non-structural protein
NV	Nanovesicle
PCR	Polymerase chain reaction
Pd-NFT	Palladium nano-thin-film
PEDOT	Poly-(3,4-ethylene dioxythiophene)
PPy	Polypyrrole
RBD	Receptor binding domain
*R*_ct_	Charge transfer resistance
RE	Reference electrode
rGO	Reduced graphene oxide
*R*_s_	Ohmic resistance
S	Spike glycoprotein
SARS-CoV	Severe acute respiratory syndrome coronavirus
SARS-CoV-2	Severe acute respiratory syndrome coronavirus 2
SELEX	Systematic evolution of ligands by exponential enrichment
SPCE	Screen-printed carbon electrode
SWV	Square wave voltammetry
TIM-1	T-cell immunoglobulin mucin domain-1
VACV	Vaccinia virus
WE	Working electrode
ZIKV	Zika virus
*Z*_w_	Warburg impedance
